# Does 3D-assisted surgery of tibial plateau fractures improve surgical and patient outcome? A systematic review of 1074 patients

**DOI:** 10.1007/s00068-021-01773-2

**Published:** 2021-08-31

**Authors:** Nick Assink, Inge H. F. Reininga, Kaj ten Duis, Job N. Doornberg, Harm Hoekstra, Joep Kraeima, Max J. H. Witjes, Jean-Paul P. M. de Vries, Frank F. A. IJpma

**Affiliations:** 1 Department of Trauma Surgery, University of Groningen, University Medical Center Groningen, Hanzeplein 1, 9713 GZ Groningen, The Netherlands; 2 Department of Oral and Maxillofacial Surgery, 3D Lab, University of Groningen, University Medical Center Groningen, Groningen, The Netherlands; 3 Department of Traumatology, KU Leuven University Hospitals Leuven Gasthuisberg Campus, Leuven, Belgium; 4 Department of Surgery, University Medical Center Groningen, Groningen, The Netherlands

**Keywords:** Tibial plateau fracture, Three dimensional, 3D printing, Guided surgery, 3D preoperative planning, 3D virtual surgical planning

## Abstract

**Purpose:**

The aim of this systematic review was to provide an overview of current applications of 3D technologies in surgical management of tibial plateau fractures and to assess whether 3D-assisted surgery results in improved clinical outcome as compared to surgery based on conventional imaging modalities.

**Methods:**

A literature search was performed in Pubmed and Embase for articles reporting on the use of 3D techniques in operative management of tibial plateau fractures. This systematic review was performed in concordance with the PRISMA-guidelines. Methodological quality and risk of bias was assessed according to the guidelines of the McMaster Critical Appraisal. Differences in terms of operation time, blood loss, fluoroscopy frequency, intra-operative revision rates and patient-reported outcomes between 3D-assisted and conventional surgery were assessed. Data were pooled using the inverse variance weighting method in RevMan.

**Results:**

Twenty articles evaluating 948 patients treated with 3D-assisted surgery and 126 patients with conventional surgery were included. Five different concepts of 3D-assisted surgery were identified: ‘3D virtual visualization’, ‘3D printed hand-held fracture models’, ‘Pre-contouring of osteosynthesis plates’, ‘3D printed surgical guides’, and ‘Intra-operative 3D imaging’. 3D-assisted surgery resulted in reduced operation time (104.7 vs. 126.4 min; *P* < 0.01), less blood loss (241 ml vs. 306 ml; *P* < 0.01), decreased frequency of fluoroscopy (5.8 vs. 9.1 times; *P* < 0.01). No differences in functional outcome was found (Hospital for Special Surgery Knee-Rating Scale: 88.6 vs. 82.8; *P* = 0.23).

**Conclusions:**

Five concepts of 3D-assisted surgical management of tibial plateau fractures emerged over the last decade. These include 3D virtual fracture visualization, 3D-printed hand-held fracture models for surgical planning, 3D-printed models for pre-contouring of osteosynthesis plates, 3D-printed surgical guides, and intra-operative 3D imaging. 3D-assisted surgery may have a positive effect on operation time, blood loss, and fluoroscopy frequency.

**Supplementary Information:**

The online version contains supplementary material available at 10.1007/s00068-021-01773-2.

## Introduction

Intra-articular fractures of the tibial plateau are usually composed of complex fracture patterns including multiple fracture fragments, which are displaced and rotated in multiple directions. Achieving normal knee alignment and an optimal reconstruction of the articular surface decreases the risk of progressive osteoarthritis [1]. However, due to the complexity of these fractures, the goals of surgery cannot always be achieved. Recently, it has been shown that up to 30% of the surgically treated tibial plateau fractures resulted in a suboptimal reduction [2]. Assessment of the fracture is essential to fully understand the fracture pattern and to choose the optimal treatment strategy. Clinical decision-making and preoperative planning is mostly based on conventional imaging modalities, including plain radiographs, two-dimensional (2D) fluoroscopy and 2D CT images [3]. With these modalities, it is difficult to fully comprehend the true extent of these injuries, since the fracture fragments are often displaced and rotated in multiple directions. 3D visualization and printing modalities have the potential to provide the physician with a better understanding of the fracture pattern and could improve treatment strategy and patient outcome [4, 5].

The growing popularity and expansion across industries providing 3D printing resources has substantially decreased costs, increased access, and led to multiple applications in orthopaedic trauma surgery [6, 7]. Early results on the clinical application of 3D printing improved levels of understanding into complex fractures for both surgeons and patients and strengthened the informed consent process [8]. Also, 3D technologies may be valuable for teaching students about fracture morphology or explaining residents about the surgical plan [9]. 3D-assisted surgery encompasses the use of 3D technology to pre-plan the operation and guide the surgeon to the planned outcome during surgery. This includes a spectrum of modalities such as 3D visualization, 3D printing and patient-specific surgical guides or implants. However, the potential advantages of 3D-assisted surgery in tibial plateau fracture management are still subject of debate.

Despite the rapid advances in technology and an increasing number of publications on the applications of 3D technologies, a comprehensive overview of the current evidence for the application of 3D-assisted surgery of tibial plateau fractures is still lacking. Therefore, the purpose of this systematic review is to provide a complete and comprehensive overview of the currently used concepts of 3D-assisted surgery in patients receiving surgical treatment for their tibial plateau fracture by including both observational and intervention studies. The aim is to answer the following clinical research questions: (1) Does the clinical application of 3D-assisted surgery for tibial plateau fractures improve intra-operative results in terms of operation time, blood loss, fluoroscopy time and intra-operative surgical revisions compared to conventional surgery? (2) Does the application of 3D-assisted surgery improve postoperative results in terms of patient functional outcome compared to conventional surgery?

## Materials and methods

This systematic review was performed according to the Preferred Reporting Items for Systematic Reviews (PRISMA) [10]. The protocol of this systematic review is registered in the international PROSPERO-database (CRD42021235524). Ethical approval was not required for this study.

### Search strategy

The Pubmed and Embase libraries were searched on the 1st of February 2021 for articles published on state-of-the-art 3D technology between January 2010 until January 2021. The search string was developed in collaboration with a medical librarian. The exact search string for the different libraries is shown in the online supplementary (Appendix 1 in Supplementary file 1).

### Study selection

Eligible studies for inclusion were randomized controlled trials, prospective and retrospective observational studies, descriptive studies, and case reports reporting on the use of 3D techniques in the management of tibial plateau fractures in orthopaedic trauma patients. Studies were excluded in case of: (1) paediatric fractures; (2) fracture classification studies; (3) animal or cadaveric studies; (4) review articles, letters to the editor or conference abstracts; and (5) studies in another language than English, German, French, Spanish or Dutch.

All articles were imported into Rayyan QCRI, a web-based sorting tool for systematic literature reviews [11]. The study selection was then performed in two phases: first two reviewers (NA, FIJ) independently screened the articles for eligibility based on the titles and abstracts using the Rayyan QCRI tool. Second, all articles which were considered eligible, were subsequently screened in full text by the same reviewers. Disagreement was resolved by discussion according to the Cochrane Handbook for Systematic Reviews of Interventions [12].

### Quality check and data extraction

Methodological quality and risk of bias of the included studies were independently assessed by NA and FIJ according to the guidelines of the McMaster University Occupational Therapy Evidence-Based Practice Research Group [13]. Any continued disagreements were solved during a consensus meeting with NA, FIJ and IR. The McMaster critical appraisal consists of eight categories including: (1) study purpose; (2) literature review; (3) study design; (4) study sample; (5) study outcome; (6) study intervention; (7) study results; and (8) conclusions and implications. Scores were giving with ‘yes = 1 point’, ‘no = 0 points’, ‘not addressed (NS)’, and ‘not applicable (NA)’. The total score reflects the methodological quality with a maximum score of 16 for RCTs and 14 for other designs. The definitive score is expressed as a percentage that may vary from 0 to 100%, with a higher score indicating a higher methodological quality. Scores between 90 and 100% were considered as excellent quality, studies between 75 and 89% as good quality studies and studies < 75% as moderate quality studies.

The data extraction was independently conducted (NA, FIJ) using a precompiled extraction file (Microsoft Excel version 14.0; Microsoft Inc., Redmond, WA, USA). Information on study characteristics, fracture classification, 3D technologies and outcome measures were extracted. In case data regarding the reported outcomes was missing, authors were contacted to retrieve raw data or means with their standard deviations.

### Outcome measures

All parameters describing the operation were determined to assess the effect of 3D-assisted surgery on intra-operative results. These parameters include operation time, blood loss, fluoroscopy time, and the number of intra-operative revisions of the fracture reduction or implant position as a result of intra-operative 3D imaging. Second, Patient-Reported Outcome Measures (PROMs) were recorded to evaluate the effect of 3D-assisted surgery on postoperative functional outcome.

### Statistical analysis

Analysis of the extracted data was performed using RevMan (version 5.4.1). Continuous variables were presented as means with standard deviation (SD) and dichotomous variables as frequencies and percentages. Continuous outcomes were pooled using the inverse variance weighting method and were presented as weighted mean difference (WMD) with the corresponding 95% confidence interval (95%CI). Heterogeneity between studies was assessed for all reported outcomes by the I^2^ statistic for heterogeneity. The I^2^ statistic was interpreted according to the benchmarks of the Cochrane Handbook for Systematic Reviews of Interventions, which considered < 40% as irrelevant, 30–60% as moderate heterogeneity, 50–90% as substantial heterogeneity, and > 75% as considerable heterogeneity [12]. A *P *value of < 0.05 was considered to indicate statistical significance.

## Results

### Search

**Fig. 1:**
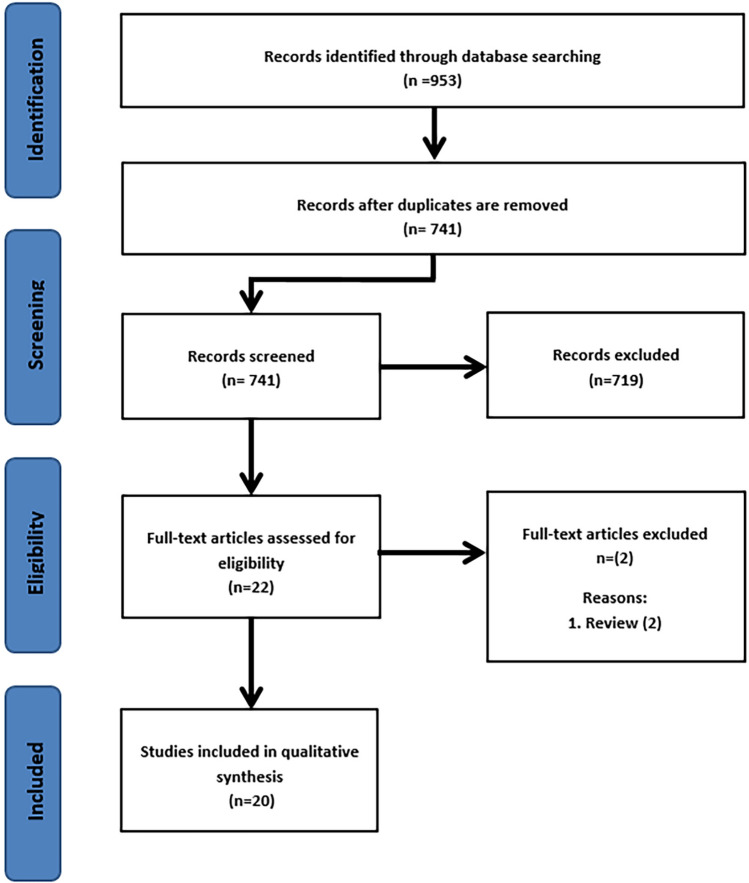
Flow diagram according to PRISMA strategy

The search resulted in 953 studies, and after removal of duplicates, 741 eligible studies were screened on title and abstract. Eventually, 22 articles were included for full-text screening of which two articles were excluded [14, 15]. Twenty studies met the inclusion criteria of this systematic review [8, 16–34]. The review process is summarized in Fig. 1. There were seven prospective cohort studies [20, 21, 24, 27, 28, 32, 34], four retrospective cohort studies [16, 19, 26, 29], five case series [22, 23, 30, 31, 33], two case reports [17, 18], one descriptive study [8], and one observational study [25]. No Randomized controlled trials were found. The included studies enrolled a total of 1074 patients with a tibial plateau fracture (mean sample size 53.7; 1–559). Of all included patients, 948 received 3D-assisted tibial plateau fracture surgery and 126 had conventional surgery. There were no differences in fracture classifications between the 3D-assisted and the conventional group. The study characteristics are presented in Table 1.

**Table 1:** Study characteristics

Study	Year	Country	Design	*N*	Period	3D technology assessed	Fracture classification	Outcomes of interest
Beisemann et al. [16]	2019	Germany	Retrospective cohort study	559	2001–2017	Intra-operative 3D imaging	AO/OTA: 41 B1–3 and C1-3	Intra-operative revisions
Bizzotto et al.[8]	2016	Italy	Descriptive study	102 (O/W 19 TPFs)	2014–2015	3D printed fracture model	AO/OTA: 41 B1–3 and C1-3	User-experience
Citak et al. [17]	2010	Germany; USA	Case report	1	NS	Intra-operative 3D imaging	Schatzker III	User-experience; Operation time
Delcogliano et al. [18]	2020	Switzerland; Italy	Case report	1	NS	Pre-contouring of osteosynthesis plate	NS	User-experience
Franke et al. [19]	2016	Germany	Retrospective cohort study	279 (O/W 109 TPFs)	2001–2011	Intra-operative 3D imaging	AO/OTA: 41 C1–3	Intra-operative revisions
Giannetti et al. [20]	2016	Italy	Prospective cohort study	40	NS	3D printed fracture model vs. Conventional	Schatzker I–VI	Length of hospital stay; operation time; tourniquet time; blood loss; Rasmussen functional score
Guo et al. [21]	2019	China	Prospective cohort study	28	2016–2018	3D printed fracture model vs. Conventional	Schatzker II, IV–VI	Operation time; blood loss; fluoroscopy time; costs; HSS Score
Horas et al. [22]	2020	Germany	Case series	4 (O/W 1TPF)	NA	3D printed fracture model	Moore type II	User-experience
Huang et al. [23]	2018	China	Case series	6	2013–2014	3D Printed surgical guide	Schatzker V–VI	Screw length; screw entry point; screw direction
Lou et al. [24]	2016	China	Prospective cohort study	72	2014–2015	3D printed fracture model vs. Conventional	Schatzker III–VI	Operation time; blood loss; no. fluoroscopy; HSS Score
Mishra et al. [25]	2019	India	Observational study	91 (O/W 10 TPFs)	2017–2019	Pre-contouring of osteosynthesis plate	NS	Surgeons experience
Nie et al. [26]	2019	China	Retrospective cohort study	13	2015–2016	3D Printed surgical guide	Schatzker V–VI	Length of screws; Operation time; Blood loss; HSS Score
Ozturk et al. [27]	2020	Turkey	Prospective cohort study	20	2017–2018	3D printed fracture model vs. Conventional	Schatzker I, II and VI	Operation time; Blood loss; tourniquet time; no. fluoroscopy; Rasmussen score
Ruan et al. [28]	2011	China	Prospective cohort study	30	2009 – 2010	Intra-operative 3D imaging vs. conventional	Schatzker II, IV–VI	Intra-operative revisions
Shen et al. [29]	2020	China	Retrospective cohort study	42	2014–2018	3D printed fracture model vs. conventional	Schatzker IV–VI	Operation time; blood loss; no. fluoroscopy; no. plate reshaping; Rasmussen score; HSS score
Suero et al. [30]	2010	USA; Germany	Case series	5	NS	3D virtual visualization	AO/OTA: 41 B3, C1 and C3	Planning time 3D reconstruction
Wang et al. [31]	2017	China; United Kingdom	Case series	6	NS	3D printed surgical guide	Schatzker I, III and IV	Likert scale; radiographic reduction; Oxford Knee Score
Wu et al. [32]	2019	China	Prospective cohort study	69	2014–2016	3D printed fracture model	Schatzker V–VI	Radiographic reduction; Rasmussen Clinical Functional Score; Infections
Yang et al. [33]	2016	China	Case series	7	2012–2014	3D printed fracture model	Schatzker I–III	Operation time; blood loss; Rasmussen anatomy score; Rasmussen knee functional score
Zhang et al. [34]	2015	China	Prospective cohort study	36	2011–2013	3D virtual visualization vs. conventional	Schatzker III	Operation time; incision length; blood loss

*TPFs *tibial plateau fractures, *O/W *of which, *NA *not applicable, *NS *not addressed

### Identified 3D applications in tibial plateau fracture surgery

**Fig. 2:**
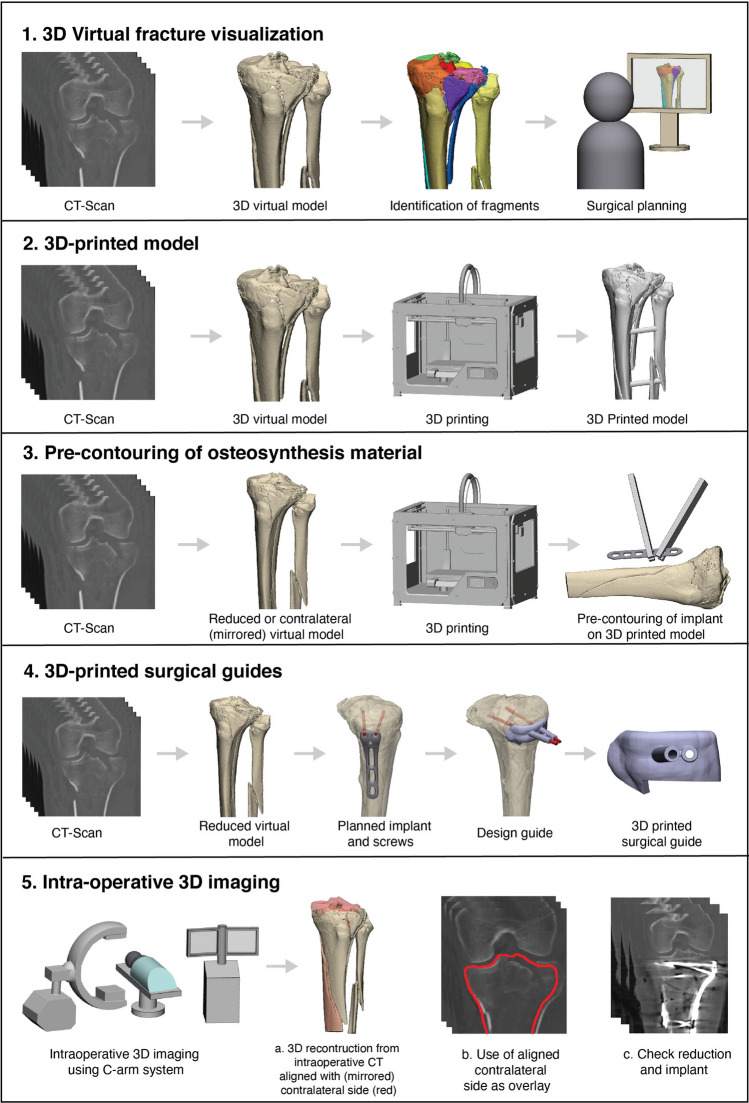
Schematic overview of the different concepts of 3D-assisted surgery in tibial plateau fractures

Within this search, five different concepts of 3D-assisted surgery in the management of tibial plateau fractures were identified over the past decade: ‘3D virtual fracture visualization’, ‘3D printed hand-held models’, ‘Pre-contouring of osteosynthesis plates’, ‘3D printed surgical guides’ and ‘Intra-operative 3D imaging’. Figure 2 depicts a representation of these concepts.

#### 3D virtual fracture visualization

Two studies reported about the use of 3D virtual visualization of the fracture before surgery [30, 34]. Suero et al. used the VoXim software (IVS Solutions AG, Chemnitz, Germany) to create a 3D reconstruction of the fracture from which the surgeon determined the surgical plan [30], whereas Zhang et al. used the Mimics software (Materialise, Leuven, Belgium) to determine a preoperative plan in which the reduction procedure was simulated [34]. Using the 3D software, the required elevation of the depressed articular surface was measured and the surgical procedure was virtually planned.

#### 3D-printed hand-held models

**Fig. 3:**
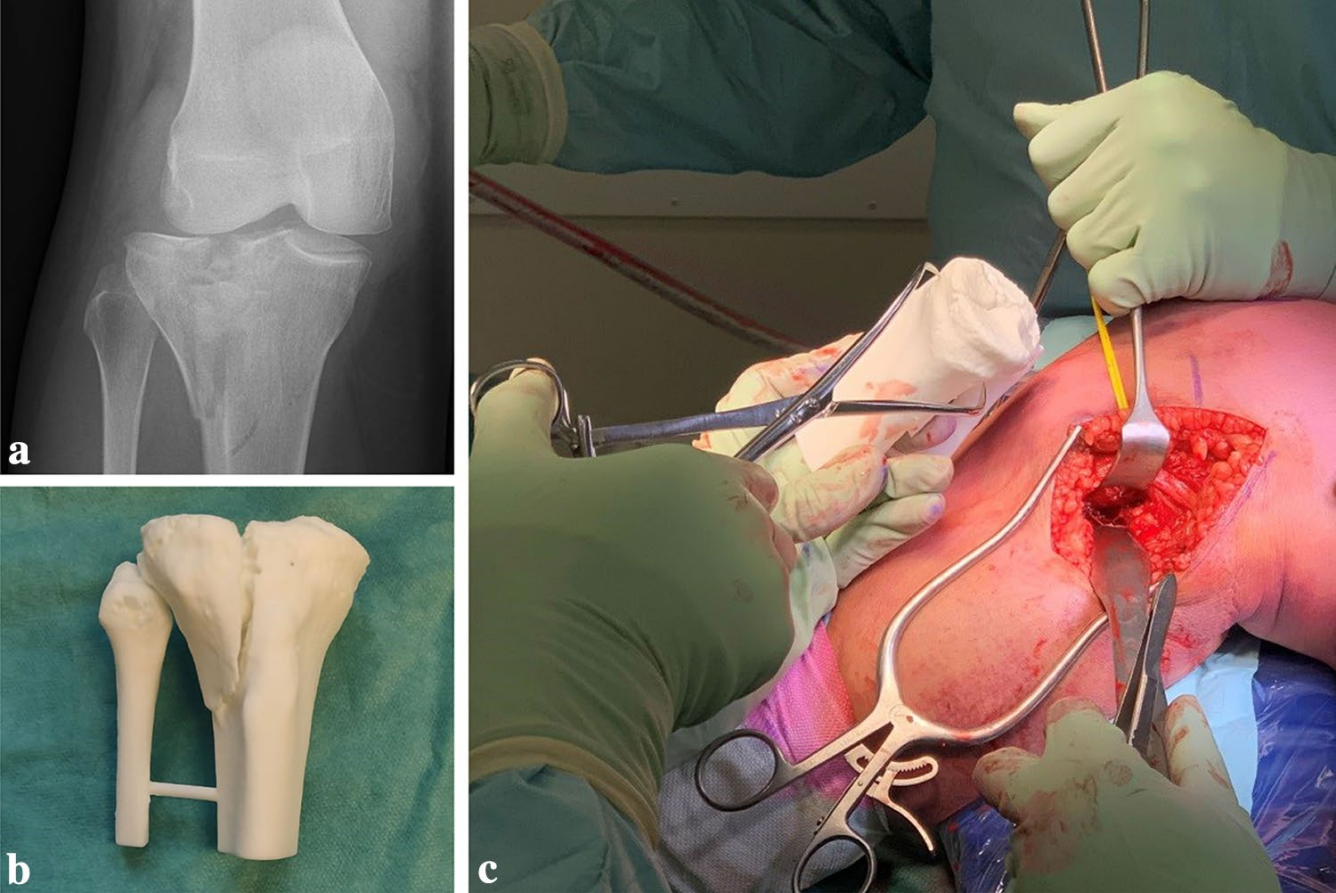
**a** Fluoroscopy of an intra-articular fracture of the tibial plateau. **b** 3D-printed han-held model of the tibial plateau fracture. **c** Intra-operative fracture assessment using the 3D-printed hand-held model

The majority of the studies reported on the use of 3D-printed models of tibial plateau fractures [8, 20–22, 24, 27, 29, 32, 33]. In these studies, a 3D-printed model of the fractured tibial plateau was used to determine the surgical plan and to guide the surgeon during surgery (Fig. 3). Furthermore, the 3D-printed models were found to be useful for educating residents and students, and to inform patients about their injury [8].

#### Pre-contouring of osteosynthesis plates

Two studies reported on the use of pre-contoured osteosynthesis plates [18, 25] using either a 3D-printed contralateral mirrored tibia or a virtually reduced fracture model. Using the printed models, implants were (pre-)operatively bended for optimized fitting along the contour of the proximal tibia.

#### 3D printed surgical guides

Three studies described the use of 3D-printed surgical guides [23, 26, 31]. In two studies, the directions of the screws were virtually predetermined. A surgical guide was designed to translate the predetermined screw trajectories to the actual surgical procedure [23, 26]. Another application of a surgical guide was found in the operative correction of a malunited tibial plateau fracture by Wang et al. [31]. First the osteotomy was performed using a guide, which helped the surgeon to perform the (virtually) predetermined osteotomy. Secondly, a reduction guide was applied to help the surgeon to reduce the fragment to its original anatomical position.

#### Intra-operative 3D imaging

Four studies reported on the use of intra-operative 3D images [16, 17, 19, 28]. These studies investigated the use of an intra-operative 3D imaging system, which was used to verify whether the achieved surgical reduction was satisfactory. Using this technology, the surgeon was able to make prompt perioperative decisions based on 3D instead of 2D fluoroscopy images. In case of dissatisfaction with the articular reduction or the position of the screws or implant, the surgeon could decide instantly during the operation to perform a revision.

### Effect of 3D-assisted surgery on clinical outcome

To answer the first research question, the effect of 3D-assisted surgery on intra-operative results in terms of operation time, blood loss, fluoroscopy time and intra-operative revisions was assessed. The second research question concerns the effect of 3D-assisted surgery on post-operative results in terms of functional outcome.

#### Operation time

**Fig. 4:**
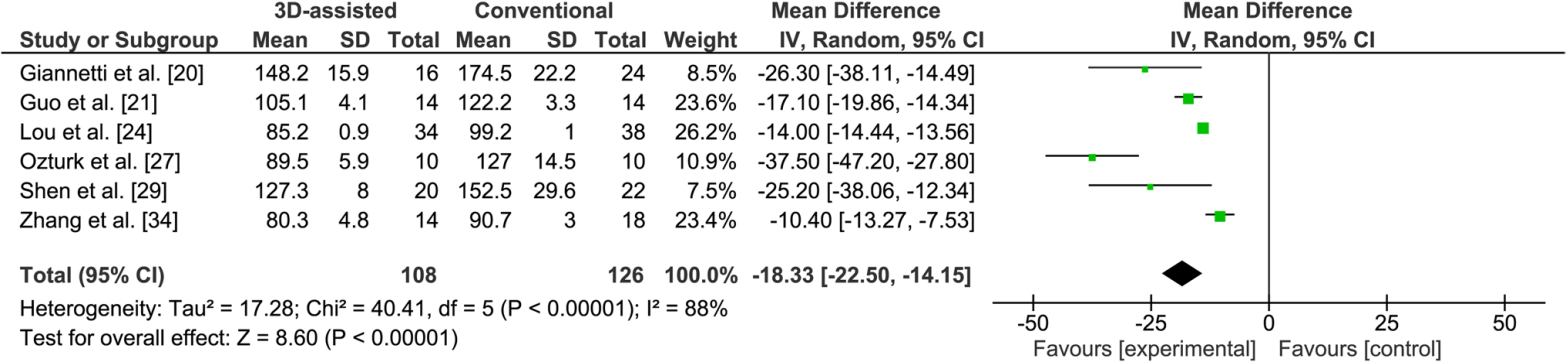
Forest plot for the operation time (min)

Six studies reported on operation time [20, 21, 24, 27, 29, 34], including one excellent quality, one good quality and four moderate quality studies (Appendix 2 in Supplementary file 2). Five studies reported that surgery assisted by a 3D-printed hand-held model of the fracture led to a significantly shorter operation time in comparison with conventional surgery [20, 21, 24, 27, 29]. Zhang et al. reported that the use of a preoperative 3D virtual model resulted in a significantly reduced operation time compared to conventional surgery [34]. The operation time was significantly shorter for the 3D-assisted group in comparison with the conventional group weighted mean difference (WMD) 18.3 min, 95% CI −22.5 to −14.5) (Fig. 4). The heterogeneity was considerable within these studies (*I*^2^ = 88%).

#### Blood loss

**Fig. 5:**
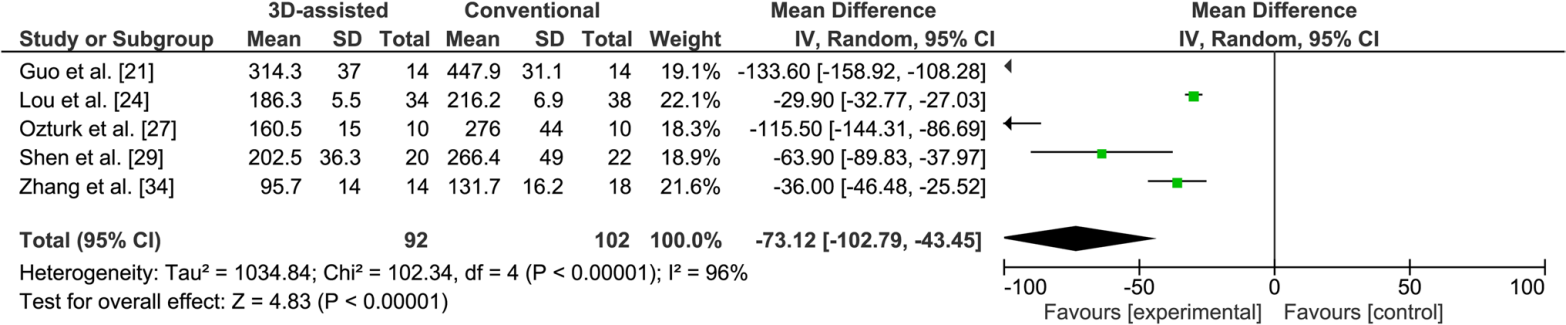
Forest plot for the blood loss (ml)

Six studies reported on blood loss [20, 21, 24, 27, 29, 34]. However, Giannetti et al. did not report the standard deviation and was, therefore, excluded from further analysis [20], leaving five studies, including one excellent quality study and four moderate quality studies. Four studies reported that 3D-printed model-assisted fracture surgery led to significantly less blood loss in comparison with conventional surgery [21, 24, 27, 29]. Zhang et al. reported that the use of a preoperative 3D virtual model resulted in significantly less blood loss compared to conventional surgery [34]. The blood loss was significantly less in the 3D-assisted group in comparison with the conventional group (WMD 73.1 ml, 95% CI −102.8 to −43.5) (Fig. 5). The heterogeneity was considerable within these studies (*I*^2^ = 96%).

#### Fluoroscopy frequency

**Fig. 6:**
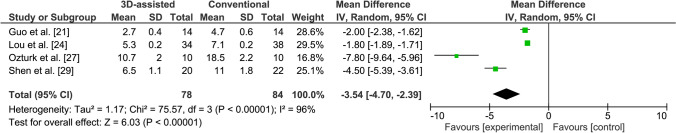
Forest plot for the fluoroscopy frequency (number of shots)

Four studies reported on the frequency of use of fluoroscopy [21, 24, 27, 29], including one study of excellent quality and three of moderate quality. The use of fluoroscopy was significantly reduced in the 3D-assisted group in comparison with the conventional group (WMD 3.5 times used, 95% CI −4.7 to −2.4) (Fig. 6). The heterogeneity was considerable within these studies (*I*^2^ = 96%).

#### Intra-operative revision rates

Three studies reported on immediate intra-operative revision rates resulting from intra-operative 3D imaging [16, 19, 28], including two good quality and one moderate study. These articles reported on a total of 698 patients of which 183 (26.2%) patients had an instant intra-operative revision of the fracture reduction or implant position as a result of intra-operative 3D imaging (Table 2).

**Table 2:** Study outcomes

Measure	Study	3D technology	Groups		Outcome		
			3D (N)	Conventional (N)	3D	Conventional	P value
**Operation results**							
**Operation time (min)**	Giannetti et al. [20]	3D printed fracture models	16	24	148.2 ± 15.9	174.5 ± 22.2	0.041*
Mean ± SD	Guo et al.[21]	3D printed fracture models	14	14	105.1 ± 4.1	122.2 ± 3.3	< 0.05*
	Lou et al.[24]	3D printed fracture models	34	38	85.2 ± 0.9	99.2 ± 1.0	< 0.001*
	Ozturk et al. [27]	3D printed fracture models	10	10	89.5 ± 5.9	127 ± 14.5	< 0.05*
	Shen et al.[29]	3D printed fracture models	20	22	127.3 ± 8.0	152.5 ± 29.6	0.001*
	Zhang et al.[34]	3D virtual visualization	14	18	80.3 ± 4.8	90.7 ± 3	< 0.001*
**Blood loss (ml)**	Guo et al.[21]	3D printed fracture models	14	14	314.3 ± 37.0	447.9 ± 31.1	< 0.05*
Mean ± SD	Lou et al.[24]	3D printed fracture models	34	38	186.3 ± 5.5	216.2 ± 6.9	0.013*
	Ozturk et al. [27]	3D printed fracture models	10	10	160.5 ± 15	276 ± 44	< 0.05*
	Shen et al.[29]	3D printed fracture models	20	22	202.5 ± 36.3	266.4 ± 49.0	0.001*
	Zhang et al.[34]	3D virtual visualization	14	18	95.7 ± 14.0	131.7 ± 16.2	< 0.001*
**Fluoroscopy frequency (number of times)**	Guo et al.[21]	3D printed fracture models	14	14	2.7 ± 0.4	4.7 ± 0.6	< 0.05*
	Lou et al.[24]	3D printed fracture models	34	38	5.3 ± 0.2	7.1 ± 0.2	< 0.001*
	Ozturk et al. [27]	3D printed fracture models	10	10	10.7 ± 2	18.5 ± 2.2	< 0.05*
	Shen et al.[29]	3D printed fracture models	20	22	6.5 ± 1.1	11 ± 1.8	0.001*
**Intra-operative revision rates resulting from 3D imaging (%)**	Beisemann et al.[16]	Intra-operative 3D imaging	559	–	148 (26.5%)	–	–
	Franke et al.[19]	Intra-operative 3D imaging	109	–	29 (27%)	–	–
	Ruan et al.[28]	Intra-operative 3D imaging	30	–	6 (20%)	–	–
**PROMs**							
Hospital for Special Knee	Lou et al.[24]	3D printed fracture models	34	38	90.0 ± 0.3	85.0 ± 0.4	< 0.001*
Surgery (HSS) score	Shen et al.[29]	3D printed fracture models	20	22	86.1 ± 7.7	79.1 ± 6.8	0.003*
Hospital for Special Knee Surgery (HSS): Excellent and good rate (%)	Guo et al.[21]	3D printed fracture models	14	14	92.9	85.7	0.54

*Significant

#### Patient-reported functional outcome

Three studies reported on functional outcome [21, 24, 29], of which one was of excellent quality. All studies used the Hospital for Special Surgery (HSS) scoring system. The HSS scoring system is based on a total of 100 points. A HSS score of ≥ 85 points is considered excellent, 70–84 points is good, 60–69 points is fair, and ≤ 59 points is poor [35]. Two studies reported the actual HSS score [24, 29], whereas another study provided the rating of the score [21]. The weighted HSS score was 88.6 (86.1–90) in the 3D-assisted group and 82.8 (79.1–85) in the conventional group. Guo et al. reported no relevant differences in HSS score between 3D printing assisted and conventional surgery [21].

## Discussion

The rationale for applying 3D technology in tibial plateau fracture surgery is that it may optimize preoperative planning, potentially improves fracture reduction and eventually benefits the patients’ recovery. This systematic review aimed to provide an overview of the current concepts of 3D-assisted tibial plateau fracture surgery and their relation to clinical outcome. The search was not limited to study design, which provides a complete overview of all 3D applications for tibial plateau fracture surgery published over the last decade. Five different concepts of 3D-assisted surgery were identified including ‘3D virtual visualization’, ‘3D printed hand-held models’, ‘Pre-contouring of osteosynthesis plates’, ‘3D printed surgical guides’, and ‘Intra-operative 3D imaging’. Pooled analysis of studies, concerning mainly the use of 3D-printed models, showed to have a positive effect on operation time, blood loss, and fluoroscopy frequency.

This review revealed that the majority of the studies (nine) used 3D-printed hand-held fracture models in clinical practice. Converting a CT-scan into a hand-held 3D-printed model could provide valuable insights for the pre-operative planning of the fracture reduction and fixation. Care should be taken regarding the soft tissue injuries which cannot be taken into account in the 3D model. These models could be sterilized and used in theatre to guide the surgeon during the operation. From an educational perspective, these models allow surgical trainees to accurately plan the surgery ahead of time, and subsequently discuss their plan with a senior. Moreover, a 3D-printed model may help in providing patient information during clinical consultation [8]. One could argue that most of these benefits could also be achieved with only 3D virtual visualization of the fracture [36]. Besides that it saves the cost of printing (€ 50 -100,- for a proximal tibia), it is instantly available and has no environmental impact. Yet, in this review only two articles were identified that described the use of a 3D virtual model for surgical planning [30, 34]. It should be noted that 3D visualization and printing itself has a learning curve, and it takes time to become familiar with the software. Virtual preoperative planning and discussing a new case may easily take up to two hours, of which a significant part is spent on the process of segmenting the CT-scan into a 3D model, virtually reducing the fracture fragments, and predetermining the implant positions.

Several of the identified 3D concepts go beyond 3D visualization and focus on translating a predetermined plan to the operative procedure itself. Pre-contouring the osteosynthesis plate on a 3D-printed model of either the mirrored contralateral side or the reduced fracture site might improve implant fitting. Implant pre-contouring showed beneficial results in acetabular fracture surgery regarding decrease in operation time and improved fracture reduction [37]. Moreover, good implant fitting in tibial plateau fracture surgery could reduce the need for elective implant removals due to optimal fitting of bulky plates. This technology was described in two of the included articles which also showed potential improvement in operation time, fracture reduction and patient outcome [18, 25]. These studies, however, were pilot studies and, therefore, limited to small case series. The full potential of this technique should therefore be further explored.

The use of 3D-printed surgical guides should be considered another 3D technique, which aims at translating a pre-operative plan to the patient [23, 26, 31]. Three case series introduced this concept for tibial plateau fractures and showed that 3D-printed guides may help the surgeon to accurately adhere to the pre-determined surgical plan. 3D-printed surgical guides are widely used in clinical practice and have been successfully applied in neurosurgery, dental surgery, spinal surgery and maxillofacial surgery [38]. In spinal surgery for instance, the use of 3D-printed drill guides led to accurate vertebral screw insertion with a mean deviation of 1.4 mm and 6.7° from the planned entry point and screw trajectory, respectively [39].

Several studies assessed the use of intra-operative 3D imaging to verify fracture reduction, implant position, and screw trajectories and lengths. These studies showed instant intra-operative revision rates up to 27% as a consequence of the 3D imaging [16, 19, 28]. However, these studies evaluated only the intra-operative acts resulting from the 3D imaging and not the clinical outcome. Downsides of this technique are the radiation exposure and increased operation time, where in more than 70% of the patients the intra-operative 3D imaging did not lead to any adjustments in the achieved surgical reduction. It should therefore be evaluated which fractures might benefit from this technique, and which not.

The main research questions concerned the effects of 3D-assisted surgery of tibial plateau fractures on intra- and postoperative outcomes. Surgery assisted by 3D visualization or prints resulted in improved intra-operative results in terms of operation time, blood loss and frequency of fluoroscopy. This is in line with previous findings regarding the use of 3D printing techniques in orthopaedic trauma fracture care [5, 38]. 3D technology provides the surgeon the ability to extensively prepare the surgery. This benefits the workflow in the operating room leading to a reduction in operation time and the frequency of fluoroscopy. A possible explanation for the decrease in blood loss could be the efficiency during the operation and a smaller incision size due to improved preoperative planning. Zhang et al. showed that the 3D-assisted group had a significant smaller incision length [34]. Studies included in this review indicate that 3D-assisted surgery might improve functional outcome. It could be hypothesized that 3D-assisted surgery leads to improved preoperative planning and eventually better reduction of the fracture. This assumption is still a matter of debate since no post-operative CTs were available in any of the studies. The effect of the 3D technique on the fracture reduction should, therefore, be further assessed.

This review has some strengths and some limitations. First, this review provides a clinically question-driven overview about the ongoing debate whether these advanced 3D technologies contribute to operation results and patient-recovery. To present a complete overview of the stare-of-the-art 3D technologies applied for tibial plateau fracture surgery we were forced to not restrict our search to solely RCTs. Inevitably, the included studies therefore encompass a wide range of study designs including case series, observational studies and retro- and prospective cohort studies. Due to the wide range of the methodological quality and heterogeneity between these studies, the pooled analysis of operation time (*I*^2^ = 88%), blood loss (*I*^2^ = 96%) and fluoroscopy frequency (*I*^2^ = 96%) should be interpret with caution. Moreover, some studies suffered from a limited sample size. Lastly, different concepts of 3D technologies were aggregated under the term “3D-assisted surgery”. However, the studies used for the pooled analysis mainly concerned the use of 3D-printed models and 3D virtual visualization. This hampers the generalizability of the results and therefore these should be interpreted with caution. High-quality randomized controlled trials for each of the 3D application are, therefore, recommended to fully explore the potential benefits of these rapid developing advanced technologies.

## Conclusion

Over the last decade, five different concepts of 3D-assisted surgical management of tibial plateau fractures emerged: ‘3D virtual visualization’, ‘3D printed hand-held models’, ‘Pre-contouring of osteosynthesis plates’, ‘3D printed surgical guides’, and ‘Intra-operative 3D imaging’. Several studies indicate that 3D-assisted surgery had a positive effect on operation time, blood loss, frequency of fluoroscopy, and functional outcome. However, 3D technologies also come with a price in preparation time and production costs (i.e. software, materials, printing devices). The potential benefits should be further investigated in high-quality studies before widespread clinical use.

## Supplementary Information

Below is the link to the electronic supplementary material.

Supplementary file1 (DOCX 14 KB)

Supplementary file2 (DOCX 17 KB)
