# Supplementary material for: Does 3D-assisted surgery of tibial plateau fractures improve surgical and patient outcome? A systematic review of 1074 patients
Source: Eur J Trauma Emerg Surg. 2021 Aug 31;48(3):1737–49. doi: 10.1007/s00068-021-01773-2 (PMC9192447; doi:10.1007/s00068-021-01773-2)
Supplement: Supplementary file 1 — Supplementary file1 (DOCX 14 KB) [file 68_2021_1773_MOESM1_ESM.docx]

**Appendix 1: Search Strategy**

| **Database** | **Search string** |
| --- | --- |
| **Pubmed (n=401)** | (3D[tiab] OR three dimension*[tiab] OR 3 dimension*[tiab] OR "Printing, Three-Dimensional"[Mesh] OR "Imaging, Three-Dimensional"[Mesh]) AND ("Tibia"[Mesh] OR "Tibial Fractures"[Mesh] OR tibial[tiab] OR tibia[tiab]) AND (fractur*[tiab] OR "Fractures, Bone"[Mesh]) AND "2010/01/01"[PDat] : "3000/12/31"[PDat] |
| **Embase (n=521)** | ('three dimensional imaging'/exp OR 'three dimensional printing'/exp OR '3d':ti,ab OR '3 dimension*':ti,ab OR 'three dimension*':ti,ab) AND ('tibia'/exp OR 'tibial plateau fracture'/exp OR tibia:ti,ab OR tibial:ti,ab) AND ('fracture'/exp OR fractur*:ti,ab) AND [embase]/lim AND [2010-2021]/py |
