# Supplementary material for: Does 3D-assisted surgery of tibial plateau fractures improve surgical and patient outcome? A systematic review of 1074 patients
Source: Eur J Trauma Emerg Surg. 2021 Aug 31;48(3):1737–49. doi: 10.1007/s00068-021-01773-2 (PMC9192447; doi:10.1007/s00068-021-01773-2)
Supplement: Supplementary file 2 — Supplementary file2 (DOCX 17 KB) [file 68_2021_1773_MOESM2_ESM.docx]

**Appendix 2: Methodological quality and risk of bias assessment**

| **Categories** | **Shen, 2020** | **Zhang, 2015** | **Giannetti,**  **2016** | **Beisemann, 2019** | **Franke, 2016** | **Huang, 2015** | **Nie, 2019** | **Lou, 2016** | **Guo, 2019** | **Ozturk,**  **2020** | **Wu, 2019** | **Yang, 2016** | **Ruan, 2011** | **Bizotto,  2016** | **Horas, 2020** | **Suero, 2010** | **Citak, 2010** | **Delcogliano**  **2020** | **Mishra,**  **2019** | **Wang, 2017** |
| --- | --- | --- | --- | --- | --- | --- | --- | --- | --- | --- | --- | --- | --- | --- | --- | --- | --- | --- | --- | --- |
| **1. Study purpose** |  |  |  |  |  |  |  |  |  |  |  |  |  |  |  |  |  |  |  |  |
| Was the study question clearly stated? | 1 | 1 | 1 | 1 | 1 | 1 | 1 | 1 | 1 | 0 | 1 | 0 | 1 | 1 | 1 | 1 | 1 | 1 | 0 | 0 |
| **2. Literature review** |  |  |  |  |  |  |  |  |  |  |  |  |  |  |  |  |  |  |  |  |
| Was relevant background literature reviewed? | 1 | 0 | 1 | 1 | 1 | 1 | 0 | 0 | 0 | 1 | 1 | 0 | 1 | 1 | 1 | 1 | 1 | 1 | 0 | 1 |
| **3. Study design** | RCS | PCS | PCS | RCS | RCS | CS | RCS | PCS | PCS | PCS | PCS | CS | PCS | DS | CS | CS | CR | CR | OS | CS |
| Was there any potential bias ? | DB | DB | DB | PB | PB | - | - | - | DB | DB | DB | - | - | SB | - | - | - | - | DB | - |
| **4. Sample** |  |  |  |  |  |  |  |  |  |  |  |  |  |  |  |  |  |  |  |  |
| Was the sample described in detail? | 1 | 1 | 1 | 1 | 1 | 1 | 1 | 1 | 1 | 1 | 1 | 1 | 1 | 1 | 1 | 1 | 1 | 0 | 1 | 1 |
| Was the sample justified? | 1 | 1 | 0 | 1 | 1 | 0 | 1 | 1 | 1 | 0 | 1 | 1 | 1 | 1 | 0 | 0 | 0 | 0 | 1 | 0 |
| Were the groups randomized? | 0 | 1 | 0 | 0 | 0 | 0 | 0 | 1 | 1 | 1 | 0 | 0 | 0 | 0 | 0 | 0 | 0 | 0 | 0 | 0 |
| Was randomizing appropriate done? | NA | 0 | NA | NA | NA | NA | NA | 0 | 0 | NS | NA | NA | NA | NA | NA | NA | NA | NA | NA | NA |
| **5. Outcomes** |  |  |  |  |  |  |  |  |  |  |  |  |  |  |  |  |  |  |  |  |
| Were the outcome measures reliable? | 1 | 1 | 1 | 1 | 1 | 1 | 1 | 1 | 1 | 1 | 0 | 1 | 0 | 0 | 0 | 1 | 0 | 0 | 0 | 0 |
| Were the outcome measures valid? | 1 | 1 | 1 | 1 | 1 | 1 | 1 | 1 | 1 | 1 | 1 | 1 | 0 | 0 | 0 | 0 | 0 | 0 | 0 | 0 |
| **6. Intervention** |  |  |  |  |  |  |  |  |  |  |  |  |  |  |  |  |  |  |  |  |
| Intervention was described in detail? | 1 | 0 | 1 | 1 | 1 | 1 | 1 | 1 | 1 | 1 | 1 | 1 | 1 | 1 | 1 | 0 | 1 | 1 | 1 | 1 |
| Contamination was avoided? | NA | 1 | 1 | NA | NA | NA | NA | 1 | 1 | 1 | NA | NA | NA | NA | NA | NA | NA | NA | NA | NA |
| Cointervention was avoided? | NA | NS | NS | NA | NA | NA | NA | NS | NS | NS | NA | NA | NA | NA | NA | NA | NA | NA | NA | NA |
| **7. Results** |  |  |  |  |  |  |  |  |  |  |  |  |  |  |  |  |  |  |  |  |
| Results were reported in terms of statistical significance? | 1 | 1 | 1 | 0 | 0 | 1 | 1 | 1 | 1 | 1 | 1 | 1 | 1 | 0 | 0 | 0 | 0 | 0 | 0 | 0 |
| Were the analysis method/s appropriate? | 1 | 1 | 1 | 1 | 1 | 1 | 0 | 1 | 0 | 0 | 0 | 0 | 0 | 0 | 0 | 0 | 0 | 0 | 0 | 0 |
| Clinical importance was reported? | 1 | 1 | 1 | 1 | 1 | 1 | 1 | 1 | 1 | 1 | 1 | 1 | 1 | 1 | 1 | 1 | 1 | 1 | 1 | 1 |
| Drop-outs were reported? | 1 | 0 | 0 | 0 | 0 | 0 | 1 | 0 | 0 | 0 | 0 | 0 | 0 | 0 | 0 | 0 | 0 | 0 | 0 | 0 |
| **8. Conclusion** |  |  |  |  |  |  |  |  |  |  |  |  |  |  |  |  |  |  |  |  |
| Conclusions were appropriate given study methods and results? | 1 | 1 | 1 | 1 | 1 | 1 | 1 | 0 | 0 | 1 | 0 | 1 | 0 | 0 | 1 | 1 | 0 | 0 | 0 | 0 |
| **Total** | **12/**  **13** | **12/**  **15** | **11/14** | **10/13** | **10/**  **13** | **10/**  **13** | **10/13** | **11/**  **15** | **10/**  **15** | **10/ 15** | **8/**  **13** | **8/**  **13** | **7/**  **13** | **6/**  **13** | **6/**  **13** | **6/**  **13** | **5/**  **13** | **4/**  **13** | **4/**  **13** | **4/**  **13** |
| **%** | **92** | **80** | **79** | **77** | **77** | **77** | **77** | **73** | **67** | **67** | **62** | **62** | **54** | **46** | **46** | **46** | **38** | **31** | **31** | **31** |

Yes = 1 point, no = 0 points, not addressed = NS, not applicable = NA, RSC = retrospective cohort, PSC = prospective cohort, DS = descriptive study, OS = observational study, CS = case series, CR = case report, SB = Selection bias, DB =Detection Bias, PB = Performance Bias
